# Improvement in the skill of CMIP6 decadal hindcasts for extreme rainfall events over the Indian summer monsoon region

**DOI:** 10.1038/s41598-023-48268-1

**Published:** 2023-12-08

**Authors:** Gopinadh Konda, Jasti S. Chowdary, C. Gnanaseelan, Anant Parekh

**Affiliations:** Indian Institute of Tropical Meteorology, Ministry of Earth Sciences, Pune, 411008 India

**Keywords:** Climate sciences, Ocean sciences

## Abstract

Decadal climate predictions have been widely used to predict the near-term climate information relevant for decision-making at multi-year timescales. In the present study, we evaluate the quality of the Coupled Model Intercomparison Project phase-6 (CMIP6) Decadal Climate Prediction Project (DCPP) hindcasts in capturing the extreme rainfall events (EREs) over the monsoon core region during Indian summer monsoon season (June–September) up to lead years 1–10. For the first time, in this study, we have used quantile mapping approach to downscale and bias correct the DCPP CMIP6 simulation/hindcast rainfall for the better representation of EREs. Detailed analysis suggests that the models in general strongly underestimate the rainfall variability over the summer monsoon region. However, after the downscaling and bias correction, the representation of rainfall variability and intensity improved multifold. The bias-corrected decadal hindcasts in fact show ~ 80% improvement in capturing the frequency, intensity, and spatial distribution of rainfall associated with the EREs. Present study brought out a downscaled DCPP product, with potential prediction skill for EREs over India. It is important to highlight that the models predict an increase in the small and medium-area EREs as compared to the large-area EREs over the monsoon core region for the decade 2019–2028.

## Introduction

The Indian subcontinent receives 80% of its annual rainfall during the boreal summer monsoon season (June–September)^1^. In this season, the Indian subcontinent experiences widespread extreme rainfall events governed by the off-shore vortices, monsoon depressions^2,3^, and mid-tropospheric cyclones^4^. It has been documented that many regions are vulnerable to severe extreme rainfall events^5,6^. The available observations indicate an increase in widespread extreme rainfall events (EREs) over central India during 1950–2015^7–9^. A significant increase in the frequency of EREs is noticed since 1980 over central India, but small-scale events do not show any trend^10^. EREs over central India are closely associated with the active phase of monsoon intraseasonal oscillations, and monsoon low-pressure systems, mainly over the Bay of Bengal (BoB)^11^. Apart from this, changes in extra-tropical circulations have strong influence on the summer EREs^12–14^. Similarly, the role of Rossby waves and teleconnection during heavy rainfall events over Pakistan, northern India, Nepal, and Tibet have been well documented^15,16^. Patwardhan et al.^17^ highlighted the role of surface thermal forcing and moist process over northwest India in triggering EREs over central India.

The general circulation models (GCMs) playing vital role in enhancing our understanding of climate systems. The GCMs are used widely to investigate the historical and future climate variability, particularly precipitation and temperature. Many stakeholders and governments utilized the outcome of these models to prepare mitigation and adaptation planning and policies. The latest release of Coupled Model Intercomparison Project (CMIP) phase 6 has witnessed a remarkable improvement in representing the present climate^18^. Consequently, numerous studies have been conducted across various regions, to understand, attribute, and/or simulate different aspects of climate systems based on the CMIP6 outputs^19–21^. Nevertheless, there are notable challenges in utilizing the model output to understand the climate variability over monsoon regions like India. Recently, the Intergovernmental Panel on Climate Change (IPCC) report projected an increase in extremes globally throughout the twenty-first century^22^. Previous studies have demonstrated that the global warming scenario might alter the statistical properties of EREs^23^ and are attributed to increased water vapour concentration in the atmosphere. Further, an increase in intense precipitation is projected under global warming conditions over large parts of the globe by CMIP phase 3 (CMIP3) models^24^, CMIP5 models^25^, and CMIP6 models^26,27^. Ha et al.^28^ examined future simulations/projections of 16 GCMs from the CMIP6 group, and found an intensification of EREs over the Indian region. The magnitude of EREs is projected to enhance globally in the twenty-first century based on the GCMs^29,30^.

Predicting the location and intensity of EREs well in advance is a challenge for all scale/range predictions including weather. Though EREs have different features based on their time and location, certain common features can be used for their prediction. In the current global warming scenario, short-term (1–10 years) climate predictions are receiving more attention in the climate community^31,32^. Apart from the CMIP historical simulations and projections, decadal experiments are also carried out for a suite of state-of-the-art GCMs to assess the forecast skill on year to year and decadal timescales. The Decadal Climate Prediction Project (DCPP), as part of World Climate Research Programme, provides a platform to understand and predict the variability in advance. Recently, many studies assessed the temperature and precipitation predictive skill of DCPP models over global and regional scales^31^. Majority of the DCPP models show high skill for the Atlantic multi-decadal variability (AMV) and air temperature over the North Atlantic Ocean up to 9 years in advance. Using correlation analysis Mehta et al.^33^ showed the significant skill of CMIP5 decadal hindcasts in representing the global and basin averaged sea surface temperature (SST) anomalies for the period 1961–2010. Pohlmann et al.^34^ investigated the Quasi-Biennial oscillation (QBO) variability in the DCPP hindcast simulations using CMIP6 forcing. They identified that the QBO representation skill is better in the updated CMIP6 forcing compared to the CMIP5 forcing. Previous studies have examined the decadal predictability of extremes such as wind storms, temperature, and precipitation^35^.

Previous studies found that the multi-model ensemble (MME) obtained based on the DCPP models from CMIP5 and CMIP6 successfully predict El Niño southern oscillation (ENSO) over a year in advance^36^. Delgado-Torres et al.^37^ reported that the CMIP6 decadal hindcast models are able to predict the land temperature and precipitation extremes for the forecast years 1–5. However, limited skill is found for the precipitation extremes compared to extreme temperature events. Many studies evaluated the representation of climate extremes by the decadal hindcast and forecast models for different regions^37–42^, but Indian subcontinent region has been generally over looked. Examining the ability of DCPP models in representing climate extremes over the Indian region is essential in the context of its potential application. In the present study, we have mainly focused on the skill of the DCPP models in demonstrating the rainfall extremes over the Indian subcontinent. High-resolution models^43^ are arguably better suited to investigate the variability in extreme rain event conditions as they can better represent the spatial scales at which such systems develop. Considering the important spatial and temporal features of EREs over India, we developed/prepared a bias-corrected dataset of daily precipitation from the DCPP hindcast models that participated in CMIP6. This is the first study to recognize the variability and frequency of EREs over India in the near future by the DCPP hindcast. Understanding the variability of extreme precipitation events, especially in the near-future, is essential. The skill of the models in representing the monsoon rainfall and extreme rainfall distribution is shown in Sect. “Results”. Section “Summary and discussion” describes the summary and discussion. Section “Methods” describes the data and methodology.

## Results

### Climatological mean state of the monsoon

**Figure 1:**
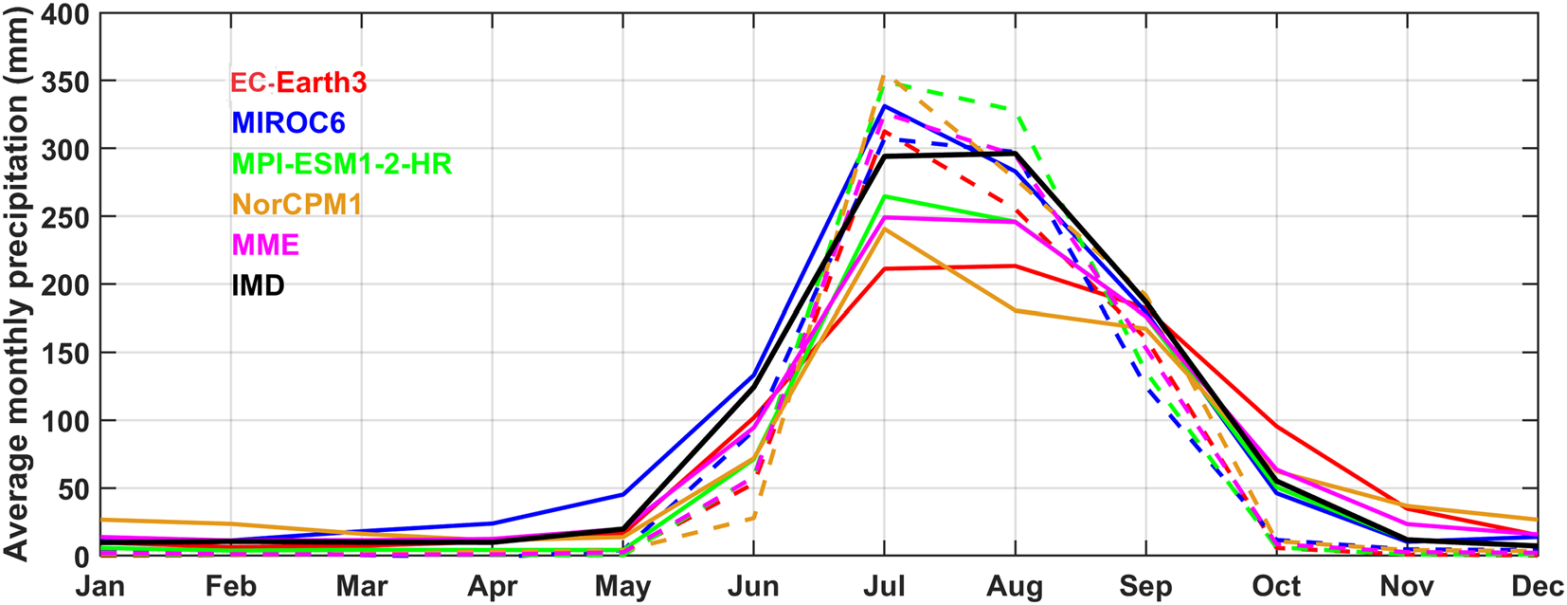
Annual cycle of monthly climatology of precipitation (mm) averaged over the Indian summer monsoon region for before (solid lines) and after (dashed lines) downscaling and bias correction (DBC) at lead year-1, black line shows observed (IMD) climatology of precipitation.

**Figure 2:**
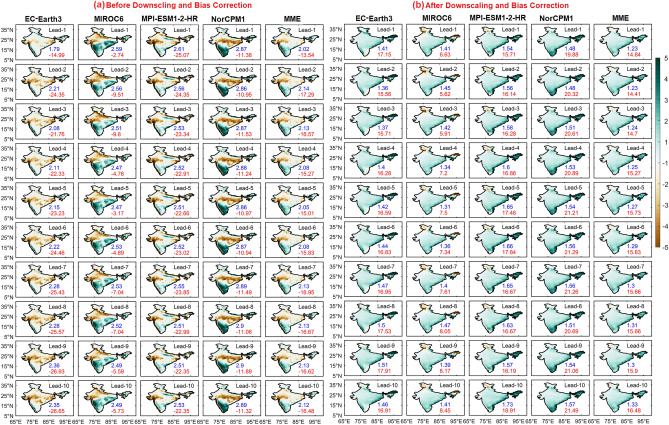
Summer monsoon seasonal mean bias of precipitation (mm/day) with lead years (from 1 to 10 top to bottom) for (**a**) before, (**b**) after DBC. Values in each panel represents the area averaged (over India) absolute mean bias (blue) and mean bias (red).

**Figure 3:**
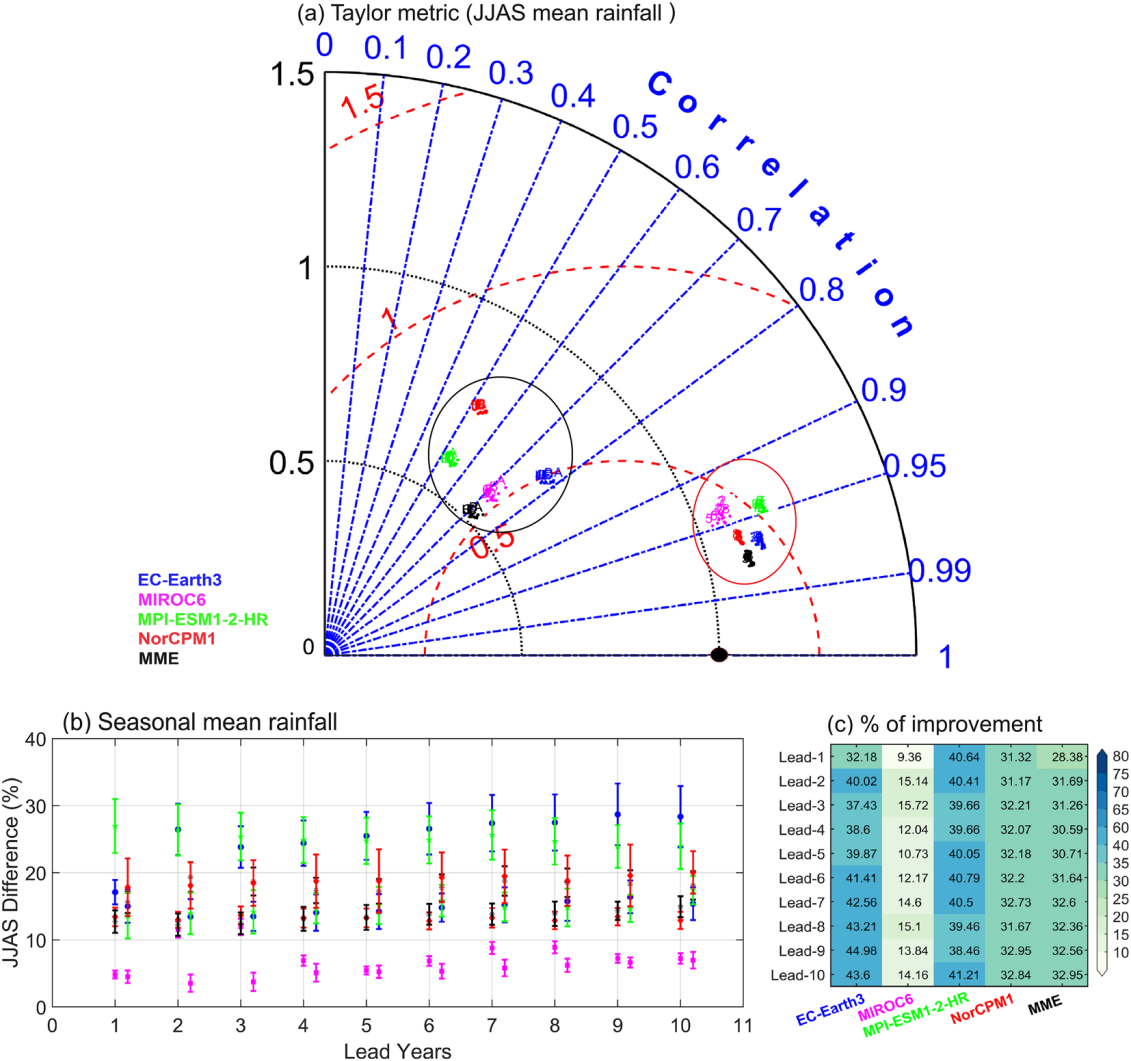
(**a**) Taylor metric for summer monsoon mean precipitation for the DCPP models (listed in Table S1). Different markers in alphabets (numerics) are for before (after) DBC with lead 1 to 10 years. The Correlation, normalized standard deviation, and normalized root mean square error are presented in blue, black and red lines respectively. Marker in black color for IMD (observations). (**b**) Difference (%) in the magnitude of JJAS [(hindcast-observation)/observation] *100 mean precipitation and its associated mean square error. Statistics before DBC lies on vertical grid lines. Statistics after DBC represented right of the vertical grid lines. (**c**) Improvement (in %) of seasonal mean rainfall over Indian summer monsoon region after DBC.

Firstly, we have analyzed the annual cycle of rainfall averaged over India for both the observations and models. Apparently, the observations show a rainfall peak in July over India^7,44,45^. Before downscale and bias correction (DBC), DCPP models, in general, at 1-year lead slightly underestimated the July rainfall peak. However, MIROC6 is able to capture the rainfall peak correctly over India, and EC-Earth3 strongly underestimated the rainfall. MME and other models (except EC-Earth3) captured the peak count well but failed to capture the magnitude of rainfall. However, after DBC, the models’ rainfall peak is close to the observations with slight overestimation (Fig. 1). MIROC6 stands out the best in the lot in representing the rainfall annual cycle. Further, we have examined the long-term mean spatial distribution of ISM rainfall over India as simulated by the DCPP models against the observations for different lead years. A comparative analysis of the models' performance in the reproducibility of the observed seasonal (JJAS) mean state of rainfall over the ISM region before and after DBC is shown in Fig. 2. Before DBC, most models (except MIROC6) showed dry bias along the monsoon trough region; however, MIROC6 model showed the dry bias over the north-western parts of India. Models also failed to simulate the rainfall over some parts of the Western Ghats (WG) and overestimated the rainfall over the southern peninsular India (east of WG) at lead-1 year. Rainfall biases in the models appear to be slightly increased with the lead time before DBC. NorCMP1 model simulated strong dry bias over monsoon trough region and also overestimated the rainfall over the foothills of the Himalayas and the north-eastern and southern parts of India (Fig. 2). After DBC, models’ dry bias over the monsoon trough region and wet bias over southern peninsular India reduced significantly (by ~ 60%), and are evident in mean and absolute bias (Fig. 3c). MME closely approximates rainfall to the observations over most of the ISM region after DBC (Fig. 2). Mean precipitation bias is found to be significantly high before DBC and low after DBC. Compared to the individual models, MME shows weak bias in precipitation over India and the bias in the models increases with increasing lead time (Fig. S1). By analyzing CMIP6 historical simulations, Konda et al.^46^ found that, the wet and dry biases in the models are due to the misrepresentation of moisture transport from the adjacent oceans and the misrepresentation of the thermodynamic process.

We have assessed the skill of DCPP models for each lead year before and after DBC in representing the mean spatial pattern of ISM rainfall over India, as shown in Fig. 3. Taylor metric indicates the significant improvement in the skill of the models after DBC for all lead predictions/years (Fig. 3a). Detailed analysis suggests that EC-Earth3 and MIROC6 models displayed good skill in representing the monsoon rainfall over India, whereas models such as MPI-ESM1-2-HR and NorCPM1 have failed. The mean error and the percentage departure of ISM rainfall is presented in Fig. 3b. It is found that the mean error and the seasonal departure of rainfall are high in the models except MIROC6 before DBC. On the other hand, MIROC6 and MME show good skill for all lead years. It is important to note that after DBC, the seasonal mean errors in models have reduced considerably and are closer to the observations (Fig. 2). The percentage improvement of ISM rainfall after DBC with lead years is shown in Fig. 3c. It is seen that the model simulated ISM rainfall over India is increased by about 30% in EC-Earth3, MPI-ESM1-2-HR, and NorCPM1 models. Consistent with the observations, DCPP models simulated the increasing trends of rainfall over the ISM region after the1990’s, with a significant increase in rainfall projected over India by 2028 (Figure not shown).

### Climatological mean features of EREs

**Figure 4:**
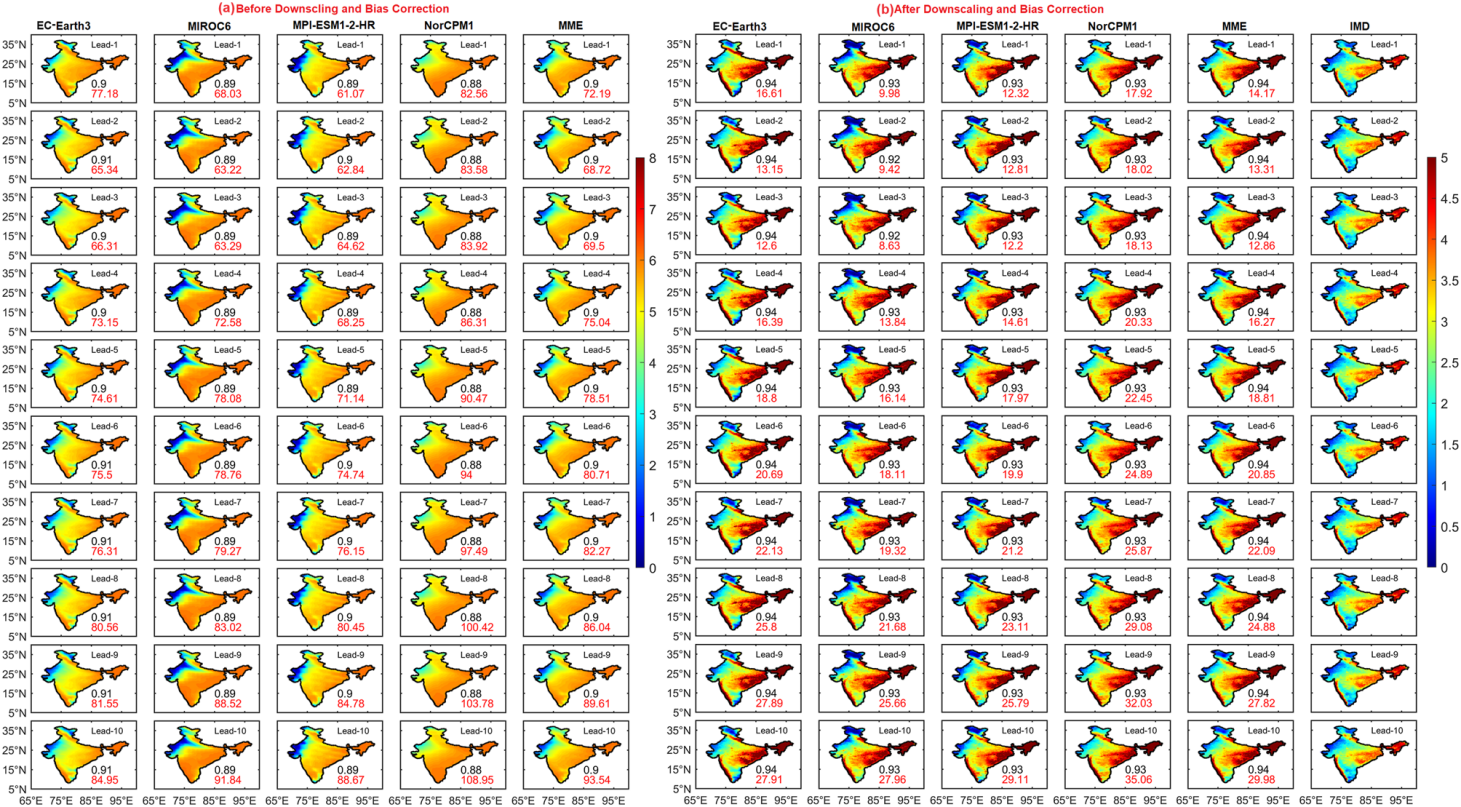
Frequency of extreme rainfall days with lead years (from 1 to 10 top to bottom) for (**a**) before, (**b**) after DBC. Values in each panel represents the percentage change in frequency distribution of extreme rainfall days (red) and pattern correlation (black).

The spatial distribution of R95 values over India suggests that the extreme rainfall threshold over WG, eastern parts of India, and central India is nearly 140, 120, and 70 mm/day, respectively, in the observations. The frequency of extreme rainfall distribution at each grid point is shown in Fig. 4. In the observations, eastern, and central India, and WG receive frequent EREs, and northwestern and south peninsular India receive less number of EREs. Before DBC, DCPP models captured the frequency distribution over the northwestern parts of India but failed to capture over southern peninsular India. It is important to note that the individual models and MME could represent the spatial distribution of EREs well after DBC, as indicated by pattern correlation ranging from 0.92 to 0.94, at all lead years. We found that, the percentage difference in EREs is high before DBC, however, this difference is minimized after DBC. This suggests the utility of DBC in DCPP models’ hindcasts generated in the study to assess the predictability of EREs. With increasing lead years, the difference in EREs distribution is escalated before and after DBC (Fig. S2).

**Figure 5:**
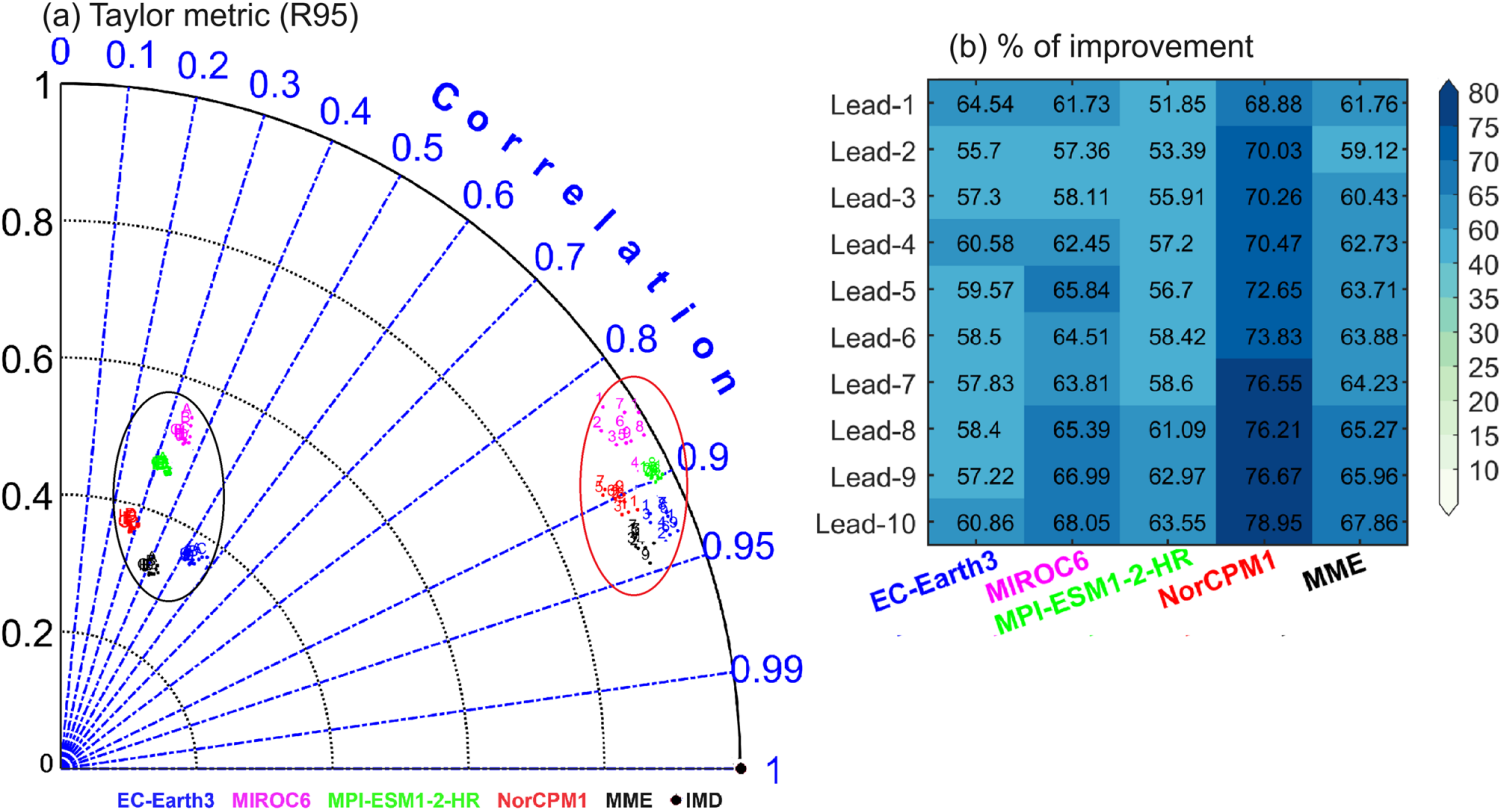
(**a**) Taylor metric for the R95 thresholds over India for DCPP models (listed in Table S1). The Correlation and, normalized standard deviation are presented in blue and black lines respectively. Different markers in alphabets (numerics) are for before (after) DBC with lead 1 to 10 years. Marker in black color for IMD (observations). (**b**) Improvement (in %) of R95 threshold distribution over Indian summer monsoon region after DBC.

Further, the skill of the models in representing the R95 thresholds averaged over India is shown in the Taylor metric (Fig. 5). The analysis suggests that, before DBC, models underestimated the R95 thresholds over India; however, it has improved significantly in all lead years after DBC (Fig. 5). Compared to the individual models, MME better captured the R95 spatial distribution over India as indicated by higher pattern correlations and standard deviation. The improvement of R95 distribution by the models after DBC is shown in Fig. 5b. It is found that, after DBC, the R95 distribution is improved by ~ 60%, and the highest improvement is seen in NorCPM1.

**Figure 6:**
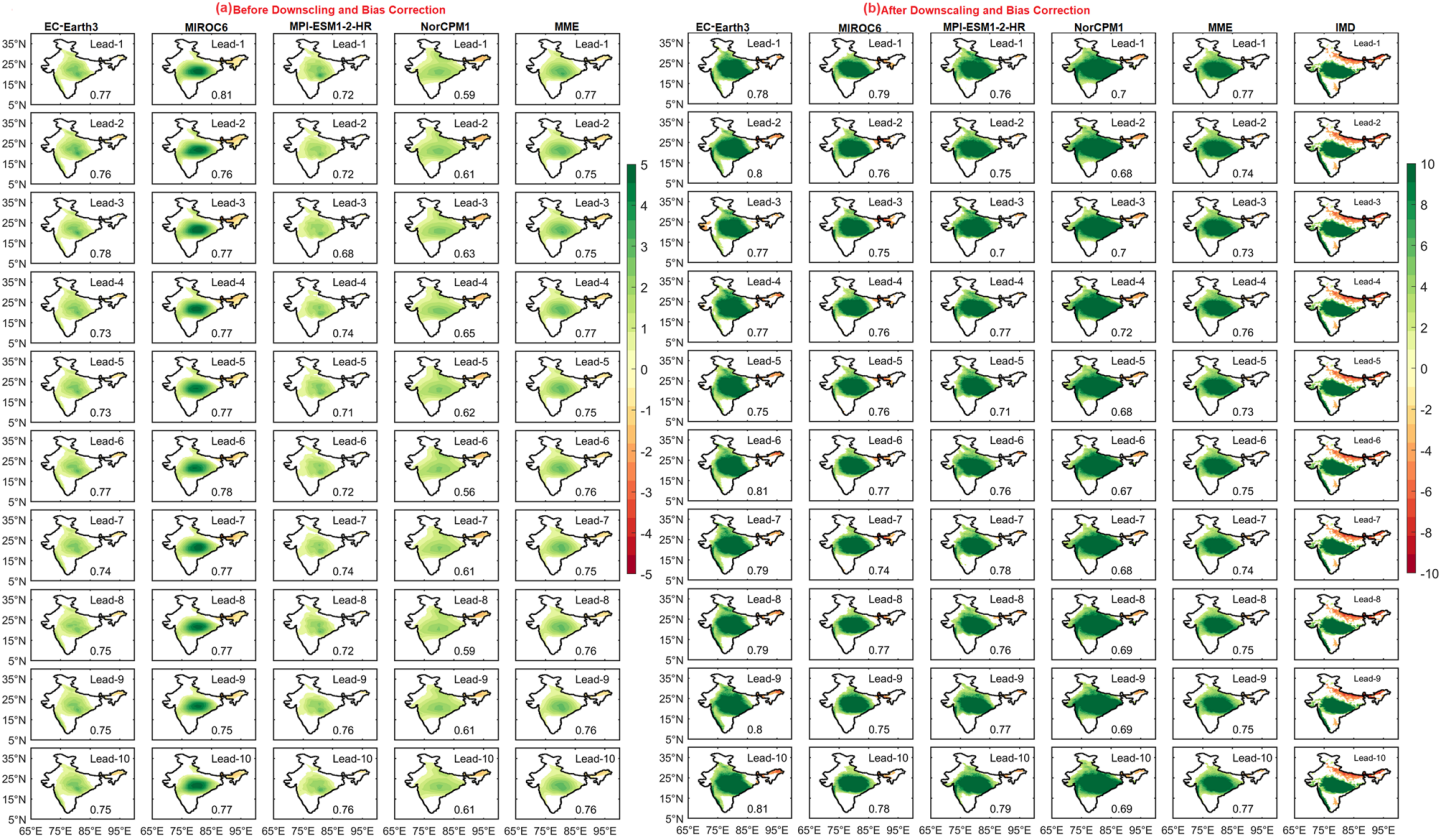
Composite of precipitation anomalies (mm/day) for large area extreme rainfall events with lead years (from 1 to 10 top to bottom) in DCPP models (**a**) before, (**b**) after DBC, and IMD. Values in each panel represents the pattern correlation (black).

**Figure 7:**
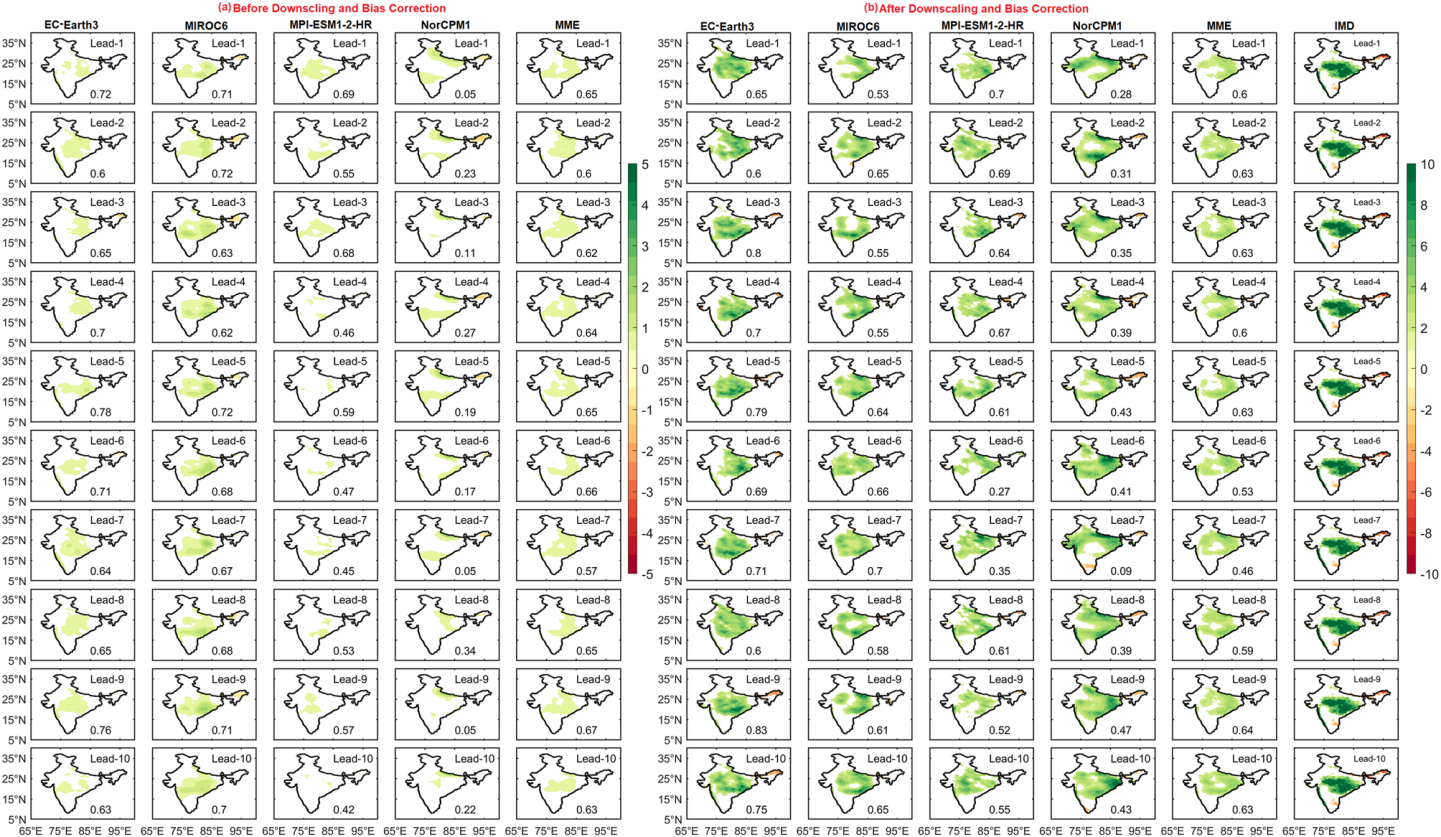
Composite of precipitation anomalies (mm/day) for small area extreme rainfall events with lead years (from 1 to 10 top to bottom) in DCPP models (**a**) before, (**b**) after DBC, and IMD. Values in each panel represents the pattern correlation (black).

In the recent years, India has been (increasingly) experiencing widespread floods induced by large-scale EREs^7,9^, which are mostly associated with the monsoon depressions^2,3,11^. Forecasting these high-flood potential events in advance is a challenge. In the present study we highlight the ability of DCPP models in representing the large-area EREs associated with monsoon depressions. The large-area EREs (> 70,000 km^2^) occur mainly over the regions of the frequent passage of synoptic systems over India's east and west coasts. Models are able to capture the rainfall patterns associated with the large EREs (composites), however, the magnitude is strongly underestimated before DBC (Fig. 6). In the observations, positive anomalies of rainfall associated with large-area EREs are seen over the monsoon core region and are majorly governed by the passage of monsoon depressions^2,3,11^. MIROC6 captured the rainfall peak associated with the EREs with more intensity compared to other models. It is important to highlight that after DBC; all the models have improved the representation of rainfall distribution associated with EREs at all lead years. However, models failed to capture the negative anomalies over the foot-hills of the Himalayas. The overestimation/underestimation of the extremes by the model might not necessarily be wrong/incorrect considering the uncertainty in the observational data over the study area especially for the extreme precipitation^47^. Increasing resolution leads to a better depiction of orography and land surface fields which are crucial for the initiation of convection in multifaceted terrain^45^, and also provides a better understanding of future climate change at smaller scale and for high-impact extreme conditions. NorCPM1 model shows a widespread distribution of convective rainfall associated with the EREs over central India. On the other hand, MME captured the observed rainfall distribution well during the large EREs. After DBC, representation of large-area EREs improved by about 80% in magnitude. Similar composite analysis is carried out for medium (10,000 km^2^ ≤ area ≤ 70,000 km^2^; Fig. S3) and small (< 10,000 km^2^; Fig. 7) area EREs.

Medium-area EREs are characterized by strong convective rainfall over the monsoon core region and the WG in the observations. DCPP models show the widespread rainfall with strong underestimation in magnitude before DBC, however the magnitude of rainfall is significantly improved after DBC by about 55%. The MIROC6 model captured the peak centers of rainfall associated with the EREs (Fig. S3). For the small-area EREs, the convection is seen mainly over east of the central Indian region. These prominent features are well captured by the DCPP models and MME after DBC (Fig. 7). The DCPP models underestimate the magnitude of the rainfall anomalies before DBC; however, the rainfall distribution and the magnitudes are well captured by the DCPP models after DBC (Fig. 7). Improvement of rainfall over the ISM region associated with the EREs is shown in Fig. S5a–c. During large-area EREs (Fig. S5a), EC-Earth3, MPI-ESM1-2-HR, and NorCPM1 models show more than 100% improvement in rainfall distribution, however, the improvement of rainfall is about 45–90% for medium and small-area EREs. This clearly suggests that, compared to the medium and small-area EREs, large-area EREs are increased by about 30% after DBC in DCPP models.

**Figure 8:**
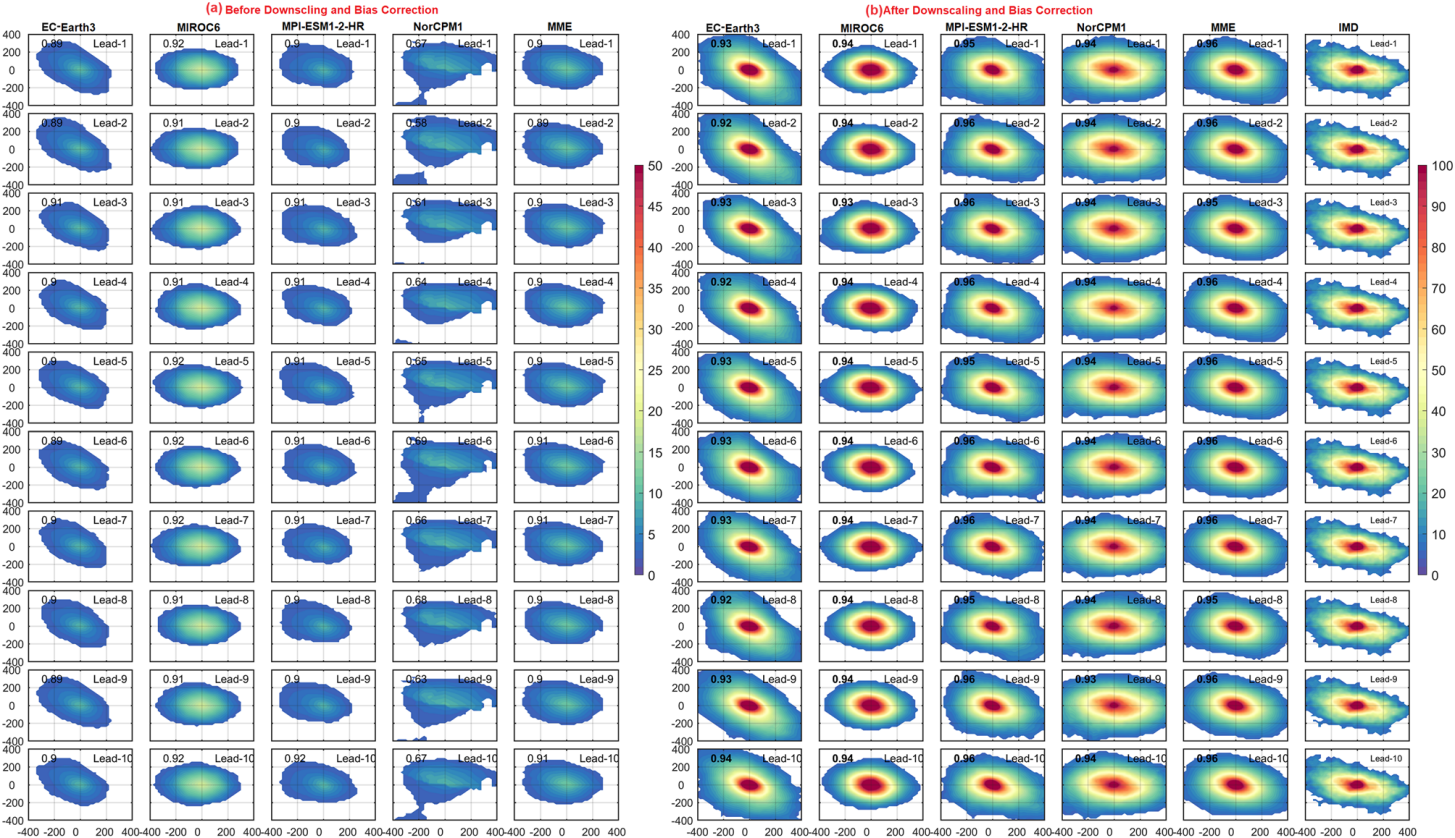
Radial distribution of precipitation (mm/day) for large area extreme rainfall events with lead years (from 1 to 10 top to bottom) in DCPP models (**a**) before, (**b**) after DBC. Values in each panel represents the pattern correlation (black).

**Figure 9:**
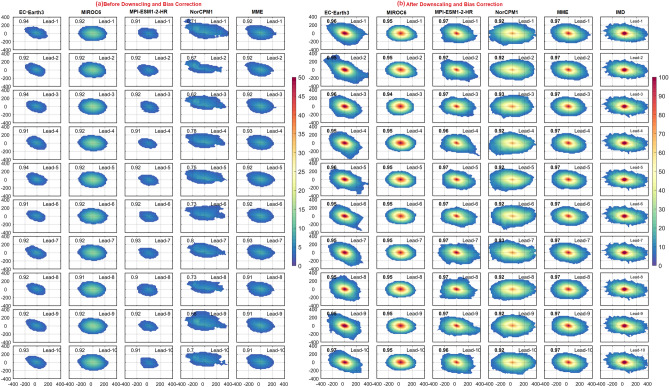
Radial distribution of precipitation (mm/day) for small area extreme rainfall events with lead years (from 1 to 10 top to bottom) in DCPP models (**a**) before, (**b**) after DBC. Values in each panel represents the pattern correlation (black).

**Figure 10:**
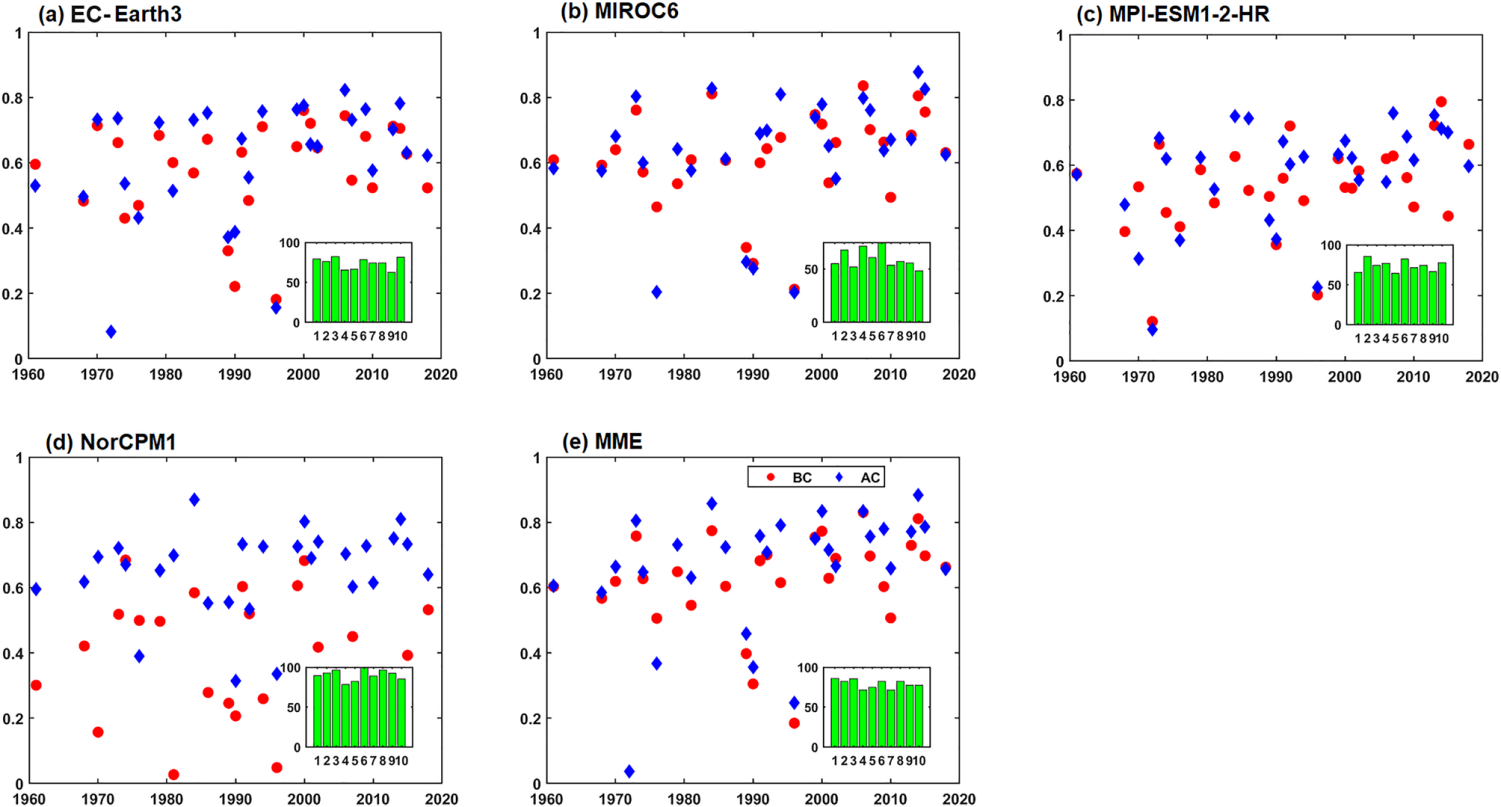
Pattern correlation of radial distribution of precipitation during large area EREs: before (red circles) and after (blue diamond) DBC in the models and MME for lead-1 year. Bar diagram inside of each panel represents the % of years having high pattern correlation after bias correction (x-axis represents the lead years and y-axis represents the % of years).

Understanding the rainfall distribution and orientation of the peak convection center is essential during the occurrence of EREs. The radial distribution of rainfall surrounded by the maxima during the large EREs before and after DBC is shown in Fig. 8. In the observations, rainfall distribution exhibits northwest-northeast orientation. Heavy rainfall within 100 km radius is found around the center of the large ERE. The DCPP models could capture the orientation of rainfall associated with the EREs but strongly underestimate the magnitude of the rainfall before DBC. Nevertheless, NorCPM1 failed to represent rainfall distribution around the peak convection center. EC-Earth3 on the other hand overestimates the tilt of the rainfall distribution. However, the rainfall distribution after DBC is closer to the observation in all models and MME at all the lead years (Fig. 8). Overall, after DBC, the representation of rainfall distribution associated with large-area EREs in DCPP models improved by 80%. On the other hand, during medium EREs, the rainfall is situated around 50 km from the center/peak (Fig. S4), whereas it is limited to ~ 30 km in small-area EREs (Fig. 9). The skill of rainfall distribution improved in all the models after DBC in both medium and small-area EREs (Figs. 9 and S4). The large-area EREs are not only widespread in nature but also more intense. The observations show that the average rainfall at the center of large, medium, and small-area EREs is ~ 150, ~ 120, and ~ 100 mm/day, respectively. After DBC, all the models (except NorCPM1) and MME captured the rainfall thresholds. Improvement of radial distribution of rainfall associated with the EREs is shown in Fig. S5d–f. During large-area EREs (Fig. S5d), models show more than 80% improvement in the radial distribution of rainfall. Consistent with the EREs rainfall distribution over India, the radial distribution of rainfall also improved after DBC. The skill of models in representing the radial distribution of rainfall for the large-area EREs at lead-1 year is shown in Fig. 10. The pattern correlation (models and observations) analysis suggests that models after DBC improved the representation of the radial distribution of rainfall. Before DBC, the NorCPM1 model showed an insignificant correlation. However, the rainfall distribution improved after DBC. It is clear that after DBC, the skill of the models in representing area-wise EREs improved significantly in most of the years. However, MME shows greater skill compared to the individual models. This study demonstrates that representation of climatological features such as mean rainfall and EREs over the ISM region is realistic after DBC.

**Figure 11:**
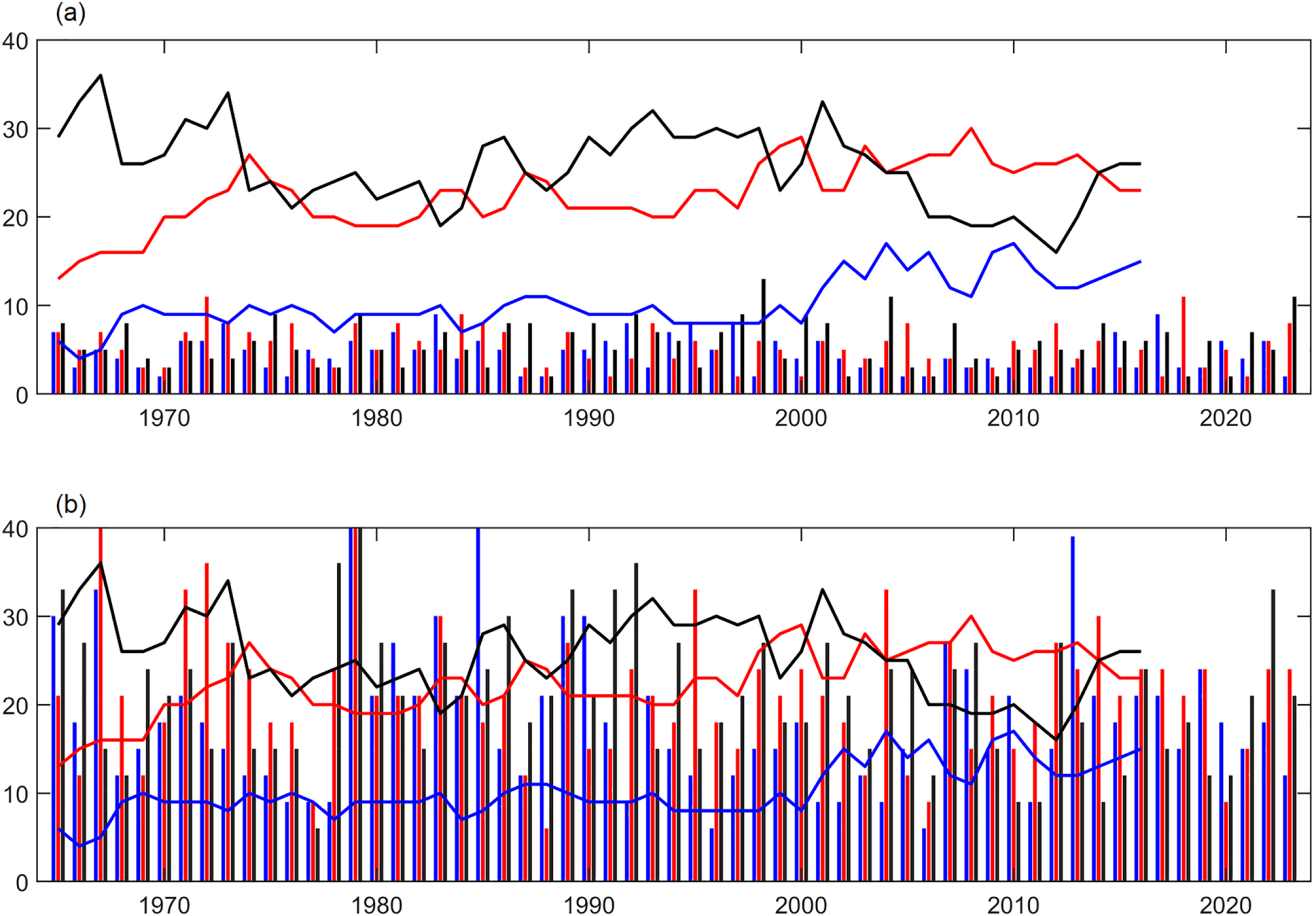
Number of extreme days over the monsoon core region for 10-year-average from 1965 to 2023 in MME, (**a**) before DBC and (**b**) after DBC, blue bars for large area, red for medium area, and black for small area extreme days. Similarly, lines represent the extreme days in the observations.

Note that the decadal predictions considered here are initiated from 1960 onwards up to 2018. The ensemble means of the 10-year average from (1961–1970) to (2019–2028), representing the years 1965–2022 over the ISM region contains decadal information. For instance, the year 1965 (2023) is used to represent the time average of data from 1961 to 1970 (2019–2028). The number of extreme days over the ISM core region for the 10-year-average (above mentioned) from 1965 to 2023 is shown in Fig. 11. On average, 10-large, 21-medium, and 26-small-area EREs are detected in the observations. DCPP models underestimated the number of extreme days over the monsoon core region compared to the observations before DBC (Fig. 11a). However, after DBC, the number of EREs over the monsoon core region during ISM season improved considerably in each decade. DCPP models projected an increase in the small and medium EREs compared to the large-area EREs over the monsoon core region for the present decade. This highlights the DCPP models’ potential in capturing the near-term regional and global climate predictions.

## Summary and discussion

Advance prediction of EREs is essential for weather/climate forecasters to reduce local calamity and water resource management. Decadal Climate Prediction Project (DCPP) models’ hindcasts are useful to assess and understand the decadal prediction of weather and climate. In particular, we have evaluated the hindcast skill/quality of the CMIP6 decadal prediction system in representing the summer monsoon (JJAS) rainfall climatology and EREs using the daily precipitation data over the Indian region for the different lead years. In this study, we applied bi-linear interpolation and Empirical Quantile Mapping to downscale and bias-correct the precipitation over India. The downscaling technique is applied to the available 4 DCPP models’ hindcasts (daily). It is found that the skill of the models in representing mean precipitation and EREs (R95) spatial distribution with lead years is higher over the Indian region after DBC, however, before DBC the skill of the models is low. It is noted that after DBC, R95 spatial distribution is improved by about 60%. Compared to the individual models, MME performed well, captured the seasonal mean and climatological features of EREs. Before DBC, models underestimate the temporal variability of monsoon rainfall and after DBC, variability is comparable to the observations. It is found that the mean state of rainfall, frequency, and extremes showed significant improvement after DBC, whereas before DBC, the models underestimated them.

In the observations, the maximum rainfall associated with large-area EREs is about 150 mm/day within 100 km radius. It is about 120 (100) mm/day with a radius of 50 (30) km for medium (small) area EREs. After DBC, the rainfall threshold is about 150 mm/day at the center of the EREs in MME and all the individual models (except NorCPM1). All models captured the characteristic features of the EREs over India after DBC for all the lead years. Greater improvement in mean rainfall distribution, frequency, and intensity of EREs is seen in DCPP models after DBC. DCPP models also project an increase in small and medium-area EREs as compared to the large-area EREs over the monsoon core regions for the decade 2019–2028. This study strongly advocates that downscaled and bias-corrected DCPP models’ hindcast would be very useful for the assessment of near-term climate change and for planning and mitigation efforts across the Indian region. These downscaled bias corrected data provide useful, more accurate depictions of extreme rainfall events. In conclusion, the DCPP CMIP6 models can skillfully capture the EREs and the distribution of rainfall associated with the EREs after the bias correction. These findings have large societal applications and are useful for climate scientists, forecasters, and policymakers.

## Methods

### Data

The observations used in the present study are gridded daily rainfall from the India Meteorological Department (IMD) available at a spatial resolution of 0.25° × 0.25° for the period 1961–2021^48^; (https://imdpune.gov.in/Clim_Pred_LRF_New/Grided_Data_Download.html). Available daily precipitation hindcasts with 10 ensemble members from CMIP6^18^ are used in the present study to examine the skill of four DCPP models in assessing the climatological features of EREs over India during boreal summer season (June to September). The CMIP6 group of models provide DCPP hindcasts for the next 10 years from initialized year (called lead-1, lead-2,…, lead-10). In this DCPP experiment, the models initialized in the month of November every year during 1960 to 2018. For example, model that is initiated in November 1960, hindcast/forecast is produced for the period January 1961 to December 1970. The first two months (Nov–Dec 1960) are considered as spin-up period of model. Hindcast/forecast of 1961 is considered as the lead-1 year, 1962 is considered as the lead-2 year etc. for the model initialized in November 1960. The same approach is followed for all the initialized years starting from November 1960 to November 2018. Therefore, the lead-1 hindcast/forecast initialized in 2018 is 2019 and lead-10 hindcast/forecast initialized in 2018 is 2028. The details of models considered in this study are given in Table S1. These models are chosen mainly based on the availability and accessibility of decadal hindcasts of daily rainfall. Model simulations are compared against observed IMD daily rainfall from 1961 to 2022.

### Downscaling and bias correction

The present study utilizes the empirical quantile mapping (EQM) approach to bias-correct the daily precipitation hindcasts with a spatial resolution of 0.25° over the Indian summer monsoon region. The spatial resolution of the models varies from about 63 to 150 km. We re-gridded models precipitation to 0.25° to maintain consistency among them. For this, we used the bilinear interpolation method^49^. We found no significant differences in precipitation patterns after the interpolation (pattern correlation is 0.93). The interpolation method used in this study does not significantly impact the spatial distribution of climatology, biases, or annual cycle. In order to produce reliable estimates of regional and local climate impact assessment, the systematic biases in the models are then corrected using EQM. The quantile mapping method adjusts the variability with the observed using a transfer function, which could be parametric or nonparametric. In general, the transfer function can be formulated as follows^49–51^.

$${X}_{m}^{o}=f({X}_{m})$$

where ${X}_{m}^{o}$ is the bias-corrected model output. If the statistical distributions of ${X}_{m}$ and ${X}_{o}$ are known, the transformation can be written as

$${X}_{m}^{o}={F}_{0}^{-1}({F}_{m}{(X}_{m}))$$

${F}_{m}$ and ${F}_{0}$ are the cumulative distribution functions (CDFs) of ${X}_{m}$ and ${X}_{0}$ respectively.

In EQM^49^ empirical CDFs are estimated from the percentiles calculated from ${X}_{m}$ and ${X}_{0}$. As a result, EQM and its variants are applied to the precipitation even if their underlying distributions are different. We have used the normalized root mean square error, standard deviation, correlation, absolute mean bias, percentage change in bias, and mean error etc. to quantify the skill of the models in representing the mean features.

### Identification of extreme rainfall events

The present study focuses on the EREs over the monsoon core region (12–28 °N and 75–85 °E) during the summer monsoon season (June–September). Following Nikumbh et al.^10^, an ERE at each grid is identified using the 95 percentile threshold (R95)^52^. Any grid point exceeding the threshold (R95) of precipitation is termed an extreme rainfall event at that grid point. The rainfall events are further classified into large-scale area (area > 70,000 km^2^), medium-scale area (10,000 km^2^ ≤ area ≤ 70,000 km^2^), and small-scale area (area < 10,000 km^2^) events as in Nikumbh et al.^10,11^. While selecting the EREs, we adopted the iterative grid point method, in which consecutive grid points of the days that exceed the R95 threshold are only considered for analysis. It mainly takes the frequency of occurrence and synoptic signatures into account. The frequency and threshold of EREs are calculated based on the R95 at each grid point during the summer monsoon. Note that we have used observed thresholds to identify the EREs in the observations and to identify the EREs in the models, we have considered the model specified thresholds of R95.

### Supplementary Information

Supplementary Information.

## Data Availability

All data are available from the following repositories: CMIP6 decadal hindcast data from https://esgf-node.llnl.gov/search/cmip6/. IMD rainfall data from https://cdsp.imdpune.gov.in/. Computations codes and downscaled and bias correction data for CMIP6 decadal hindcasts will be available from the authors upon reasonable request.

## References

[1] Gadgil S (2003). The Indian monsoon and its variability. Annu. Rev. Earth Planet. Sci..

[2] Sikka DR (1980). Some aspects of the large scale fluctuations of summer monsoon rainfall over India in relation to fluctuations in the planetary and regional scale circulation parameters. Proc. Indian Acad. Sci.-Earth Planet. Sci..

[3] Ajayamohan RS, Merryfield WJ, Kharin VV (2010). Increasing trend of synoptic activity and its relationship with extreme rain events over central India. J. Clim..

[4] Krishnamurti TN, Hawkins RS (1970). Mid-tropospheric cyclones of the southwest monsoon. J. Appl. Meteorol. Climatol..

[5] Sharma A, Goyal MK (2018). District-level assessment of the ecohydrological resilience to hydroclimatic disturbances and its controlling factors in India. J. Hydrol..

[6] Singh YT (2019). Rapid assessment of coastal biodiversity post-2015 Chennai flood, India. EnvironmentAsia.

[7] Goswami BN (2006). Increasing trend of extreme rain events over India in a warming environment. Science.

[8] Pai, D. S. & Sridhar, L. Long term trends in the extreme rainfall events over India. In *High-Impact Weather Events Over the SAARC Region*, 229–240 (2015).

[9] Roxy MK (2017). A threefold rise in widespread extreme rain events over central India. Nat. Commun..

[10] Nikumbh AC, Chakraborty A, Bhat GS (2019). Recent spatial aggregation tendency of rainfall extremes over India. Sci. Rep..

[11] Nikumbh AC (2020). Large-scale extreme rainfall-producing synoptic systems of the Indian summer monsoon. Geophys. Res. Lett..

[12] Ramaswamy C (1958). A preliminary study of the behaviour of the Indian southwest monsoon in relation to the westerly jet stream. Geophysica.

[13] Raman CRV, Rao YP (1981). Blocking highs over Asia and monsoon droughts over India. Nature.

[14] Krishnan R (2009). Internal feedbacks from monsoon–midlatitude interactions during droughts in the Indian summer monsoon. J. Atmos. Sci..

[15] Priya P (2017). Changing monsoon and midlatitude circulation interactions over the Western Himalayas and possible links to occurrences of extreme precipitation. Clim. Dyn..

[16] Hunt KM, Turner AG, Shaffrey LC (2018). Extreme daily rainfall in Pakistan and north India: Scale interactions, mechanisms, and precursors. Mon. Weather Rev..

[17] Patwardhan, S. et al. Synoptic scale systems. In *Assessment of Climate Change Over the Indian Region: A Report of the Ministry of Earth Sciences (MoES), Government of India* (eds Krishnan, R. et al.), 143–154 (Springer Nature, 2020).

[18] Eyring V (2016). Overview of the Coupled model intercomparison project phase 6 (CMIP6) experimental design and organization. Geosci. Model Dev..

[19] Brunner L (2020). Reduced global warming from CMIP6 projections when weighting models by performance and independence. Earth Syst. Dyn..

[20] Fan X (2020). Global surface air temperatures in CMIP6: Historical performance and future changes. Environ. Res. Lett..

[21] Li H (2021). Drylands face potential threat of robust drought in the CMIP6 SSPs scenarios. Environ. Res. Lett..

[22] IPCC: Climate Change 2021: The Physical Science Basis. Contribution of Working Group I to the Sixth Assessment Report of the Intergovernmental Panel on Climate Change Cambridge University Press, Cambridge, United Kingdom and New York, In press, (2021)

[23] Janssen E (2016). Seasonal and regional variations in extreme precipitation event frequency using CMIP5. Geophys. Res. Lett..

[24] Wehner, M. et al. Atmospheric rivers in the CMIP3/5 historical and projection simulations (No. LBNL-5858E-Poster). (Lawrence Berkeley National Lab. (LBNL), 2012).

[25] Sillmann J (2013). Climate extremes indices in the CMIP5 multimodel ensemble: Part 2. Future climate projections. J. Geophys. Res. Atmos..

[26] Zhu H, Jiang Z, Li L (2021). Projection of climate extremes in China, an incremental exercise from CMIP5 to CMIP6. Sci. Bull..

[27] Gu L (2022). Global increases in compound flood-hot extreme hazards under climate warming. Geophys. Res. Lett..

[28] Ha KJ (2020). Future changes of summer monsoon characteristics and evaporative demand over Asia in CMIP6 simulations. Geophys. Res. Lett..

[29] Mishra AK (2019). Evidence of links between regional climate change and precipitation extremes over India. Weather.

[30] Shahi NK (2023). Assessment of future changes in high-impact precipitation events for India using CMIP6 models. Theor. Appl. Climatol..

[31] Kim HM, Webster PJ, Curry JA (2012). Evaluation of short-term climate change prediction in multi-model CMIP5 decadal hindcasts. Geophys. Res. Lett..

[32] Boer GJ (2016). The decadal climate prediction project (DCPP) contribution to CMIP6. Geosci. Model Dev..

[33] Mehta VM, Wang H, Mendoza K (2013). Decadal predictability of tropical basin average and global average sea surface temperatures in CMIP5 experiments with the HadCM3, GFDL-CM2. 1, NCAR-CCSM4, and MIROC5 global earth system models. Geophys. Res. Lett..

[34] Pohlmann H (2019). Realistic quasi-biennial oscillation variability in historical and decadal hindcast simulations using CMIP6 forcing. Geophys. Res. Lett..

[35] Eade R (2012). Forecasting the number of extreme daily events out to a decade ahead. J. Geophys. Res. Atmos..

[36] Choi J (2022). Seasonal-to-decadal prediction of El Niño–southern oscillation and pacific decadal oscillation. NPJ Clim. Atmos. Sci..

[37] Delgado-Torres C (2023). Multi-annual predictions of the frequency and intensity of daily temperature and precipitation extremes. Environ. Res. Lett..

[38] Hanlon HM (2013). Can a decadal forecasting system predict temperature extreme indices?. J. Clim..

[39] Hanlon HM (2015). Near-term prediction of impact-relevant extreme temperature indices. Clim. Change.

[40] Meehl GA (2014). Decadal climate prediction: An update from the trenches. Bull. Am. Meteorol. Soc..

[41] Smith DM (2019). Robust skill of decadal climate predictions. npj Clim. Atmos. Sci..

[42] Moemken J (2021). The regional MiKlip decadal prediction system for Europe: Hindcast skill for extremes and user-oriented variables. Int. J. Climatol..

[43] Shahi NK (2018). Intra-seasonal variability of the South Asian monsoon and its relationship with the Indo-Pacific sea-surface temperature in the NCEP CFSv2. Int. J. Climatol..

[44] Krishnamurti TN, Bhalme HN (1976). Oscillations of a monsoon system. Part I. Observational aspects. J. Atmos. Sci..

[45] Shukla J, Mooley DA (1987). Empirical prediction of the summer monsoon rainfall over India. Mon. Weather Rev..

[46] Konda G, Vissa NK (2023). Evaluation of CMIP6 models for simulations of surplus/deficit summer monsoon conditions over India. Clim. Dyn..

[47] Shahi NK (2022). Assessment of the spatio-temporal variability of the added value on precipitation of convection-permitting simulation over the Iberian Peninsula using the RegIPSL regional earth system model. Clim. Dyn..

[48] Pai DS (2014). Development of a new high spatial resolution (0.25× 0.25) long period (1901–2010) daily gridded rainfall data set over India and its comparison with existing data sets over the region. Mausam.

[49] Mishra V, Bhatia U, Tiwari AD (2020). Bias-corrected climate projections for South Asia from coupled model intercomparison project-6. Sci. Data.

[50] Piani C, Haerter JO, Coppola E (2010). Statistical bias correction for daily precipitation in regional climate models over Europe. Theor. Appl. Climatol..

[51] Patel J (2022). A quantile mapping approach-based bias correction in Coupled model intercomparison project phase 5 models for decadal temperature predictions over India. Int. J. Climatol..

[52] Vittal H, Karmakar S, Ghosh S (2013). Diametric changes in trends and patterns of extreme rainfall over India from pre-1950 to post-1950. Geophys. Res. Lett..

