# Supplementary material for: Improvement in the skill of CMIP6 decadal hindcasts for extreme rainfall events over the Indian summer monsoon region
Source: Sci Rep. 2023 Dec 8;13:21737. doi: 10.1038/s41598-023-48268-1 (PMC10709579; doi:10.1038/s41598-023-48268-1)
Supplement: Supplementary file 1 — Supplementary Information. [file 41598_2023_48268_MOESM1_ESM.docx]

**Improvement in the skill of CMIP6 decadal hindcasts for extreme rainfall events over the Indian summer monsoon region**

**Gopinadh Konda^1^, Jasti S. Chowdary^1^, C. Gnanaseelan^1*^, and Anant Parekh^1^**

^1^Indian Institute of Tropical Meteorology, Ministry of Earth Sciences, Pune, 411008, India

[*seelan@tropmet.res.in](mailto:*seelan@tropmet.res.in)


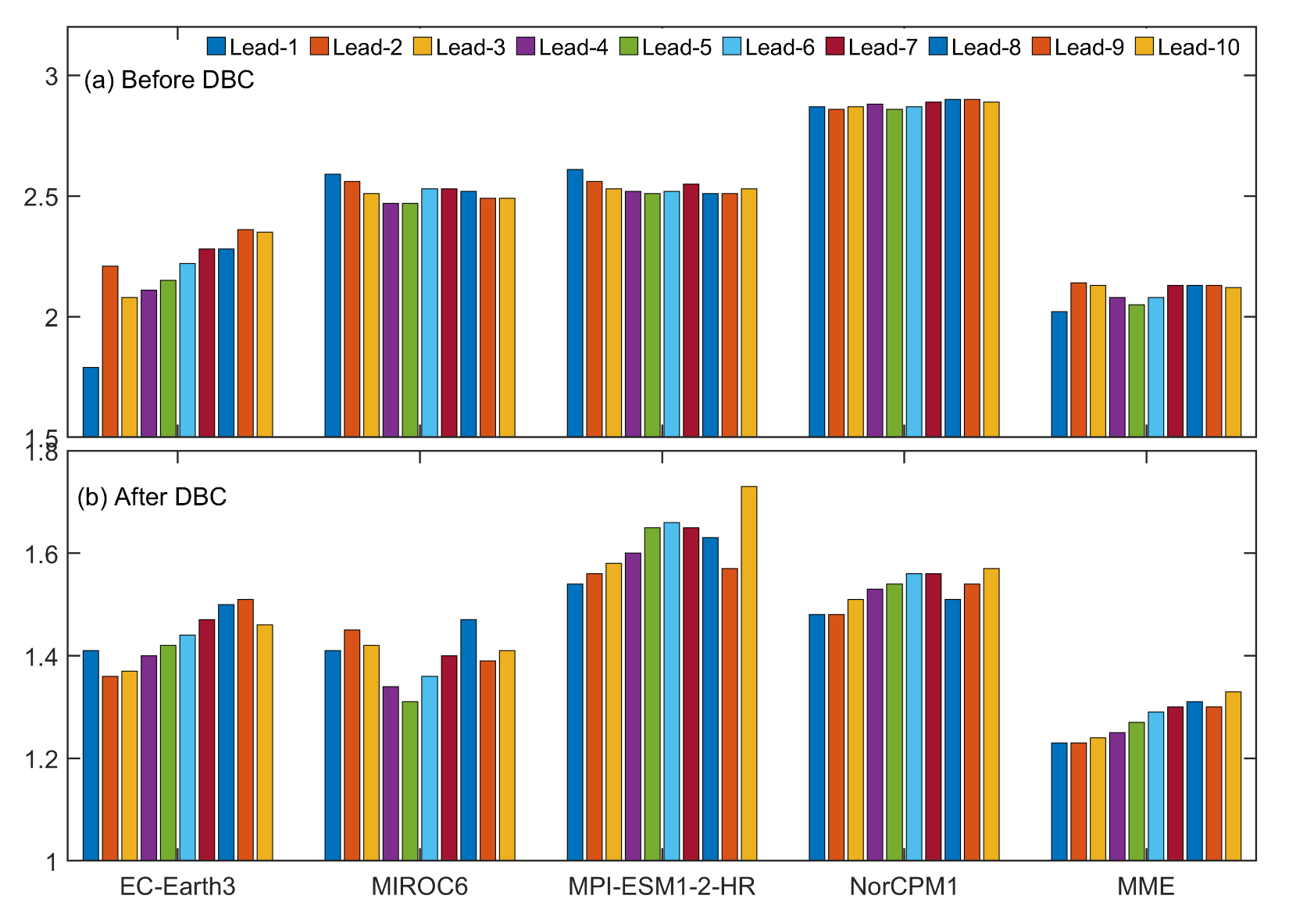


**Figure S1:** Absolute mean bias of rainfall (mm/day) averaged over India with lead years 1-10. (a) Before DBC and (b) After DBC for all the four models and also MME.


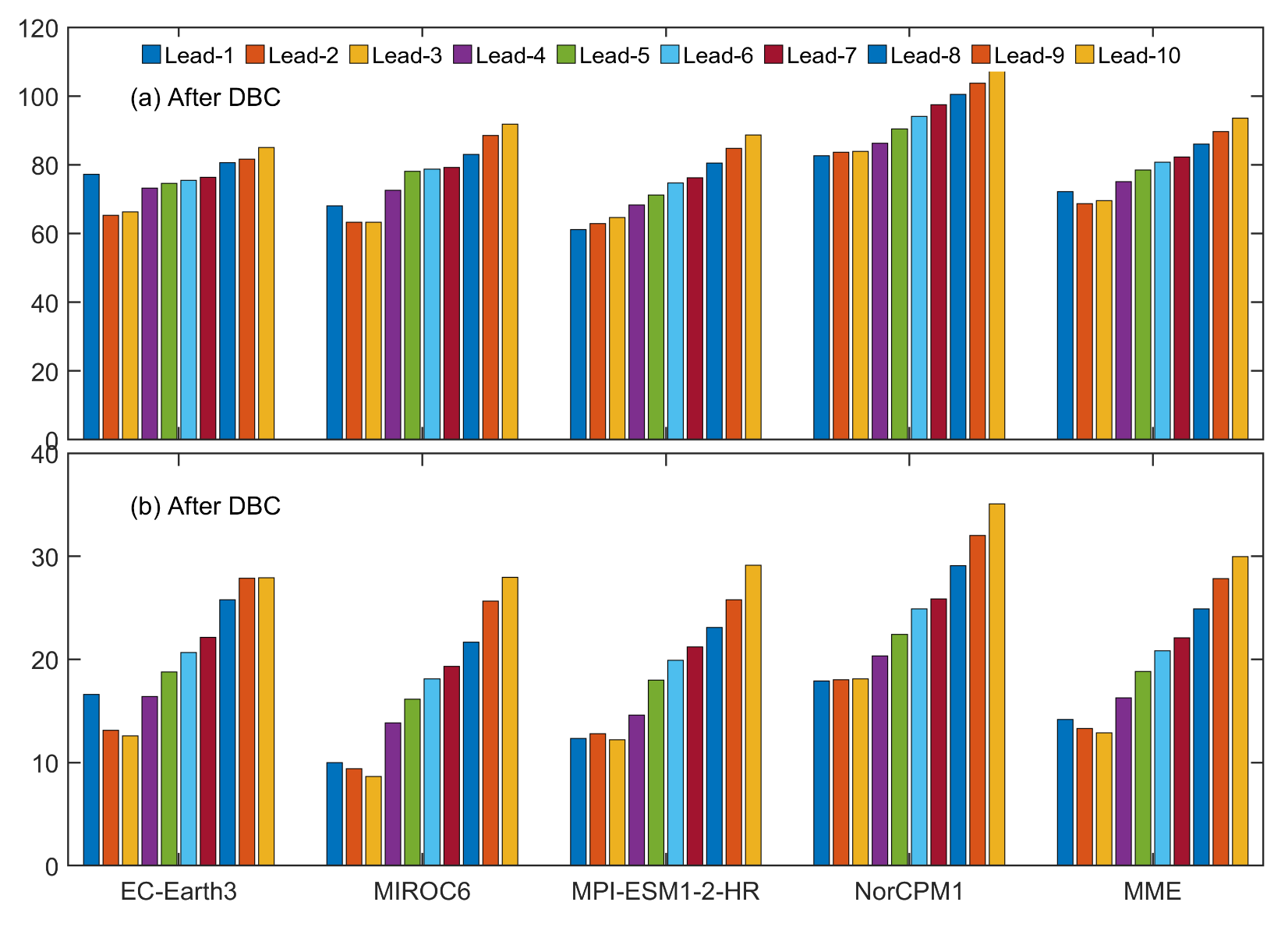


**Figure S2:** Percentage change in frequency distribution of extreme rainfall events averaged over India with lead years 1-10. (a) Before DBC and (b) After DBC for all the four models and also MME.


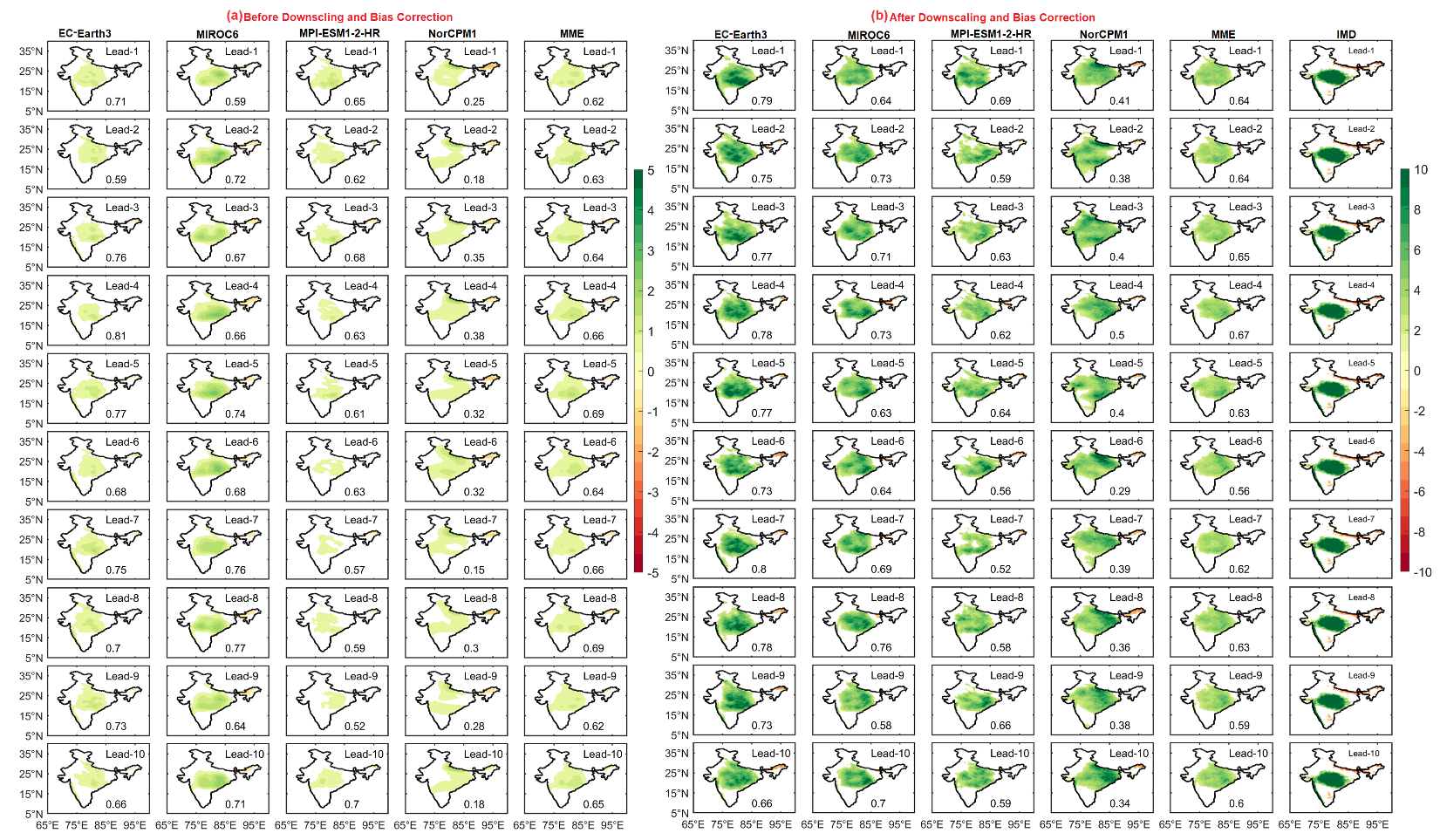


**Figure S3:** Composite of precipitation anomalies (mm/day) for medium-area extreme rainfall events with lead years (from 1 to 10 top to bottom) for (a) before, (b) after DBC. Values in each panel represents the pattern correlation (black).


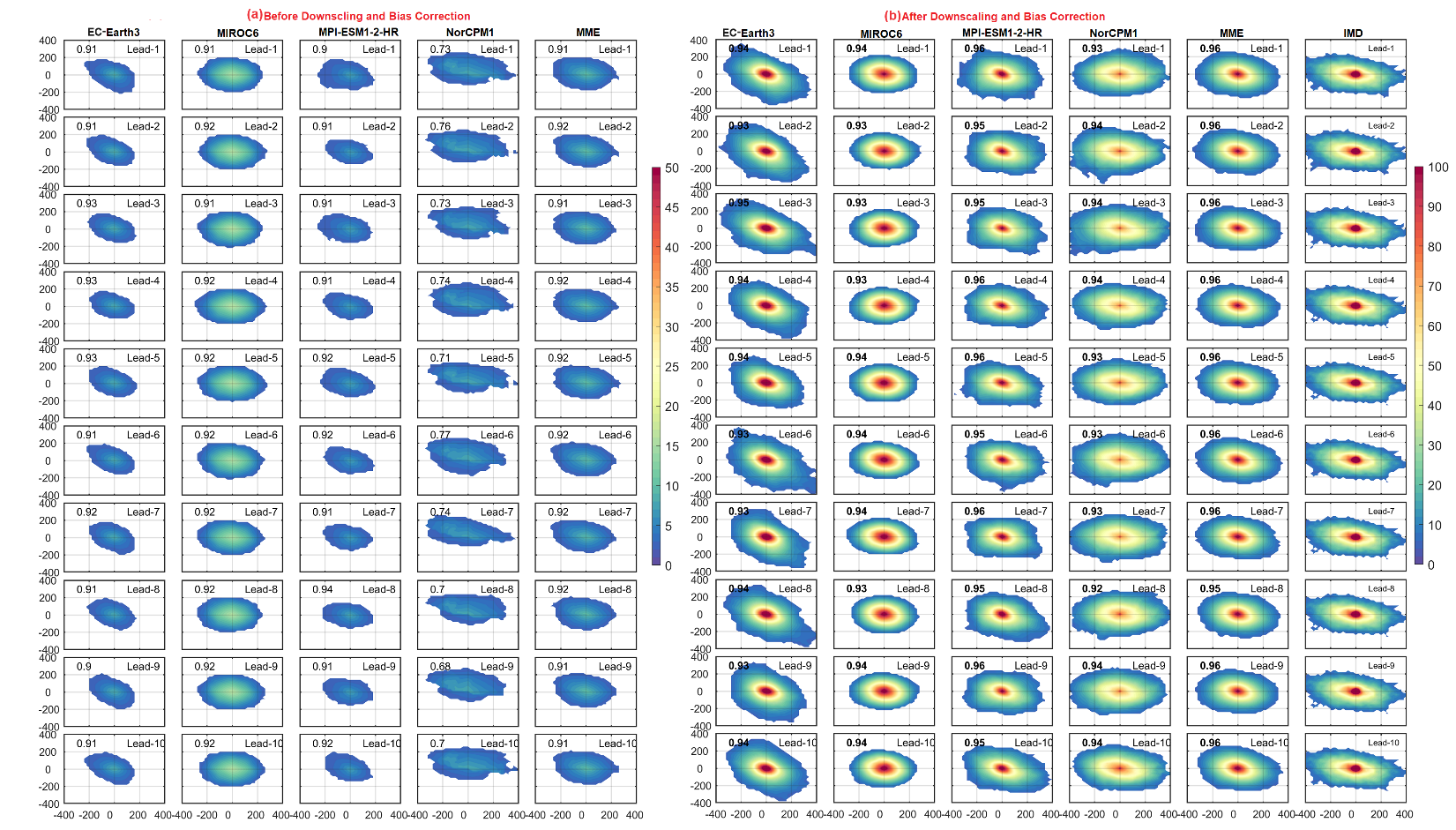


**Figure S4:** Radial distribution of precipitation (mm/day) for medium-area extreme rainfall events with lead years (from 1 to 10 top to bottom) for (a) before, (b) after DBC. Values in each panel represents the pattern correlation (black).


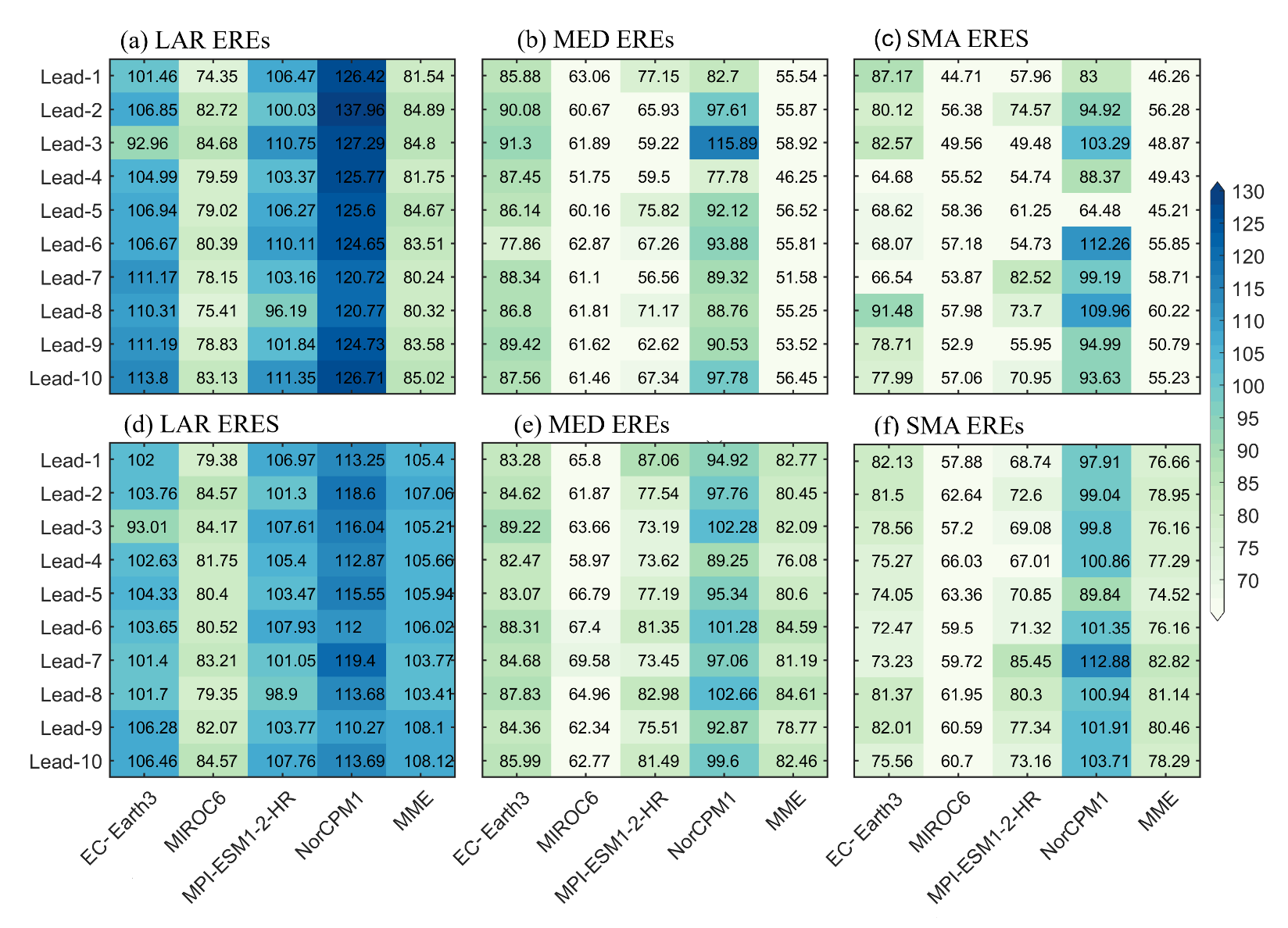


**Figure S5:** Improvement (in %) of rainfall distribution associated with the EREs over Indian summer monsoon region (top panel; a, b, c). Radial distribution of rainfall associated with the EREs over monsoon core region (bottom panel; d, e, f).

**Table S1:** Description of DCPP models used in the present study.

| **Source ID**  **&**  **Released year** | **Atmospheric model** | **Ocean model** | **Nominal**  **resolution** | **Ensemble members** | **Initial conditions (period)** |
| --- | --- | --- | --- | --- | --- |
| EC-Earth3  &  2019 | IFS cy36r4 (TL255, linearly reduced Gaussian grid equivalent to 512 x 256 lon/lat; 91 levels; top level 0.01 hPa) | NEMO3.6 (ORCA1 tripolar primarily 1 deg with meridional refinement down to 1/3 degree in the tropics; 362 x 292 lon/lat; 75 levels; top grid cell 0-1 m) | 100 km | r1i1p1f1  .  .  .  r10i1p1f1 | November  (1960-2018) |
| MIROC6  &  2017 | CCSR AGCM (T85; 256 x 128 lon/lat; 81 levels; top level 0.004 hPa) | COCO4.9 (tripolar primarily 1deg; 360 x 256 lon/lat; 63 levels; top grid cell 0-2 m) | 250 km | r1i1p1f1  .  .  .  r10i1p1f1 | November  (1960-2021) |
| MPI-ESM1-2-HR  &  2017 | ECHAM6.3 (spectral T127; 384 x 192 lon/lat; 95 levels; top level 0.01 hPa) | ECHAM6.3 (spectral T127; 384 x 192 lon/lat; 95 levels; top level 0.01 hPa) | 100 km | r1i1p1f1  .  .  .  r10i1p1f1 | November  (1960-2019) |
| NorCPM1  &  2019 | CAM-OSLO4.1 (2 degree resolution; 144 x 96 lon/lat; 26 levels; top level ~2 hPa) | MICOM1.1 (1 degree resolution; 320 x 384 lon/lat; 53 levels; top grid cell 0-2.5 m) | 250 km | r1i1p1f1  .  .  .  r10i1p1f1 | November  (1960-2018) |
